# Parent–child relationships and psychological distress: survey of parents from low-income families after the COVID-19 pandemic

**DOI:** 10.3389/fpubh.2023.1158698

**Published:** 2023-05-05

**Authors:** Li Ping Wong, Haridah Alias, Nik Daliana Nik Farid, Sofia Md Yusop, Zuhrah Musa, Zhijian Hu, Yulan Lin

**Affiliations:** ^1^Centre for Epidemiology and Evidence-Based Practice, Department of Social and Preventive Medicine, Faculty of Medicine, Universiti Malaya, Kuala Lumpur, Malaysia; ^2^Centre for Population Health (CePH), Department of Social and Preventive Medicine, Faculty of Medicine, Universiti Malaya, Kuala Lumpur, Malaysia; ^3^National Population and Family Development Board (LPPKN), LPPKN Building, Jalan Raja Laut, Kuala Lumpur, Malaysia; ^4^Department of Epidemiology and Health Statistics, School of Public Health, Fujian Medical University, Fuzhou, China

**Keywords:** youth-parent conflict, PEQ, post pandemic, B40, DASS-21

## Abstract

**Introduction:**

This study aims to shed light on parent–child relationships and the psychological health of parents from low-income families after the easing of the COVID-19 pandemic restrictions.

**Methods:**

This cross-sectional study recruited 553 parents of children aged 13–24 years in low-income community settings. The Parent–Child Conflict scale of the Parental Environment Questionnaire (PEQ) was used to measure parent–child conflict. Psychological distress was assessed using the Depression, Anxiety, and Stress Scale short form (DASS-21).

**Results:**

The study revealed a low level of parent–child conflict in the overall study population, with a median PEQ of 48.0 (interquartile range [IQR] 36 to 48). Concerning demographics, married parents reported a likelihood of having a higher level of parent–child conflict over 3 times higher than single parents (OR = 3.18 95%, CI 1.30–7.75). More parent–child conflicts were also found in parents aged 60–72 years old who were unemployed, retired, or housewives and from lower-income groups. In regard to lifestyle factors, a higher level of physical activity and having enough sleep were associated with lower levels of parent–child conflict. Only approximately 1% of the participants reported symptoms of depression, anxiety, or stress.

**Discussion:**

Low risk exists for parent–child conflict and psychological sequelae following the easing of the COVID-19 pandemic restrictions, which could be due to various support measures implemented by the government. Vulnerable parents identified as being at risk of parent–child conflict warrant attention in future advocacy efforts.

## 1. Introduction

The COVID-19 pandemic is considered the worst global health catastrophe of the century. Since it was first reported in December 2019, in Wuhan, Hubei province, China (1), the novel coronavirus rapidly spread around the world and caused enormous adverse health, economic, and social impacts on the entire human population (2). Crisis-related hardship is having a tremendous impact on parents’ and children’s psychological well-being (3, 4). The adverse psychological effect of the COVID-19 pandemic on parents and their children’s relationships is a new and unpredictable situation facing many families. The pandemic has exacerbated decreased parent–child interactions and their relational quality. Parents faced notable challenges during the COVID-19 pandemic, in particular during the period of social isolation, as they needed to juggle work and family life. As a result, negative parent–child interactions and conflicts were commonly reported during the COVID-19 pandemic (5, 6). Parent–child conflict must not be underestimated as elevated rates of conflict have been found to contribute to the development of socio-emotional dysfunction in school-age children through adolescence (7). Additionally, problematic family functioning and a negative home environment can have lasting detrimental impacts on child development (8, 9). Not only did the pressure of parenting during the COVID-19 pandemic jeopardise the well-being of children but, more importantly, such stressors may also have exerted an impact on various aspects of parents’ mental and physical health spheres; hence, primary prevention of parent–child conflict is crucial.

The impact of parent–child conflict on the physical and psychological health of parents should not be neglected. Although little has been reported on the effect of parent–child conflict on parents, pandemic-related stress in adults may have exacerbated pre-existing conditions and prospectively increased the risk of hypertension, coronary heart disease, and stroke (10). In regard to the psychological impact, there have been several reports on parenting stress affecting parental mental well-being during the COVID-19 pandemic in Asia as well as Western countries (6, 11, 12). The current evidence also suggests that the pandemic triggered an array of family issues in low-income families and economically vulnerable households (13). As parental unemployment, financial insecurity, and economic hardship caused substantial parenting stress during the COVID-19 pandemic (14, 15), parent–child conflict may have had a disproportionate impact on vulnerable parents.

It is well established that parent’s demographics are significantly related to parenting styles and children’s psychological adjustment (16). Little is known about parent’s demographics impact on parent–child relationship in the wake of the COVID-19 pandemic restrictions in Malaysia. Understanding parent–child conflict amongst a range of socioeconomic and demographic factors is important in designing equitable and appropriate support for a broad spectrum of individuals. It is also well known that the COVID-19 pandemic has negatively altered families’ lifestyles. Parents and children not meeting recommended lifestyle behaviours such as sleep time, physical activity, screen time and nutrition intake were found to be negatively associated with parent’s stress levels during the COVID-19 pandemic (17). It is uncertain whether it is possible for family to return to their normal pre-pandemic lifestyles. Establishing the relationship between lifestyle behaviours and parent–child relationship will provide insights to health authorities in designing intervention to promote healthy new normal lifestyle behaviours that foster harmonious family relationships.

In Malaysia, declining psychological and mental well-being during the COVID-19 pandemic has been reported in several studies (18–20); however, the impact of the pandemic specifically on parents and family relationships has been relatively understudied. With the pandemic broadly under control worldwide, Malaysia started relaxing its COVID-19 restrictions at the beginning of May 2022. Little is known about the parent–child relationship and whether it affects the psychological well-being of parents in low-income communities in Malaysia. It is also unknown whether the pandemic has had lasting implications for parent–child relationships after the easing of the coronavirus restrictions. Understanding the post-pandemic well-being of parents in vulnerable communities and identifying their risks and negative consequences may provide useful insights into intervening efforts. Hence, this study’s research questions focus on investigating the state of parent–child conflict and associated demographics and lifestyle factors after the government began to relax the social distancing regulations. Furthermore, this study explores the potential association between parent–child relationships and the psychological distress of parents.

## 2. Methods

### 2.1. Participants

The sample of parents in this study was recruited from residents in the People Housing Project, also known as the Programme Perumahan Rakyat (PPR), a government settlement programme for people from the low-income group (B40 or bottom 40% of the Malaysian household income) in the state of Selangor and the Federal Territory of Kuala Lumpur, Malaysia. Field enumerators were trained to recruit eligible parents and assist them in answering the survey questions. A convenience sampling approach was used. Google Surveys was used to gather data from the survey. The inclusion criteria were being parents in the PPR and having children who are in their young adulthood (aged 13 to 24 years). The questionnaire (Appendix 1) consisted of an assessment of the demographic characteristics of parents, lifestyle factors (alcohol consumption, exercise, healthy diet, and sleep quality), parent–child conflict, and psychological distress. The data collection period was from May to November 2022.

### 2.2. Assessment of parent–child conflict

The Parent–Child Conflict Scale of the Parental Environment Questionnaire (PEQ) (21 was administered to determine the respondents)’ perceptions of the parent–child relationship. The items have been shown to reliably assess five dimensions of parent–child relationships (21). Parent–child conflict refers to disharmonious or intense interactions during which both the parents and children show negative behaviours and emotions. The Parent–Child Conflict Scale consists of 12 items assessing aspects of parent–child relationship relationships on a 4-point scale (1 = definitely true, 4 = definitely false). The scores ranged from 12 to 48. All 12 items were summed, and higher overall scores reflected lower parent–child conflict. We examined the reliability of the parent–child conflict scale and the Cronbach’s α was 0.982, suggesting that the measure has a high level of internal consistency. To our best knowledge, parent–child conflict has never been assessed in the Malaysian population. In this study, the parent–child conflict scale was demonstrated to have a high internal reliability compared to other studies where the Cronbach’s α were reported to range from 0.080 to 0.090 (22–24).

### 2.3. Assessment of depression, anxiety, and stress

Psychological distress was measured using the Depression, Anxiety, and Stress Scale short form (DASS-21) (25). The scale has three subscales – namely Depression (DASS-21-D), Anxiety (DASS-21-A), and Stress (DASS-21-S). There are seven items in each subscale; the score of each subscale ranges from 0 to 21, with higher scores indicative of more severe symptoms of depression, anxiety, and/or stress. The cut-offs for depression (moderate 14–20, severe 21–27, and extremely severe ≥28), anxiety (moderate 10–14, severe 15–19, and extremely severe ≥20), and stress (moderate 19–25, severe 26–33, and extremely severe ≥34) were calculated (26). We also calculated the reliability of the subscales in this study. The Cronbach’s α for the subscales DASS-21-D, DASS-21-A, and DASS-21-S in this study was 0.975, 0.965, and 0.965, respectively, implying a high level of internal consistency. This indicate that the DASS-21 scale is a reliable psychometric instrument when used in the current study population. A former study in Malaysia reported Cronbach’s alphas of 0.956 for the overall scale, 0.927 for the DASS-21-D, 0.865 for the DASS-21-A and 0.882 for the DASS-21-S (19).

### 2.4. Statistical analyses

Scale reliability was examined using Cronbach’s alpha for internal consistency. The baseline characteristics and lifestyle factors of the study participants were summarised and categorised into two groups based on a higher and a lower score of parent–child conflict. The Kolmogorov–Smirnov and Shapiro–Wilk tests of normality were applied to understand the distribution of the data. The distribution of parent–child conflict was not normal; hence, the median (interquartile range) was used in the reporting of the results. Due to the small sample size, the association between categorical data was assessed using Fisher’s exact test. Univariate and multivariable logistic regression analyses were conducted to explore the demographics and lifestyle factors associated with parent–child conflict. Only significant factors in the univariate analyses, with a value of p of <0.05, were selected for the multivariable regression analysis. Odds ratios (OR), 95% confidence intervals (95% CI), and value of ps were calculated for each independent variable. The model fit of multivariable logistic regression analysis was assessed using the Hosmer–Lemeshow goodness-of-fit test (27). All the statistical analyses were performed using the Statistical Package for the Social Sciences version 20.0 (IBM Corp., Armonk, NY, United States).

### 2.5. Ethical considerations

This research was approved by the University of Malaya Research Ethics Committee (UM.TNC2/UMREC–1579). The respondents were informed that their participation in this research was voluntary and all consented. Data security and participants’ confidentiality were maintained at all levels of data management. Due to the sensitivity of the issue, the availability of counseling services was made known to the study participants and contact information was available to participants who needed counseling or mental health services. None of the parents reported that they were distressed or psychologically uneased by participating in the study and none used the counseling services provided.

## 3. Results

**Figure 1:**
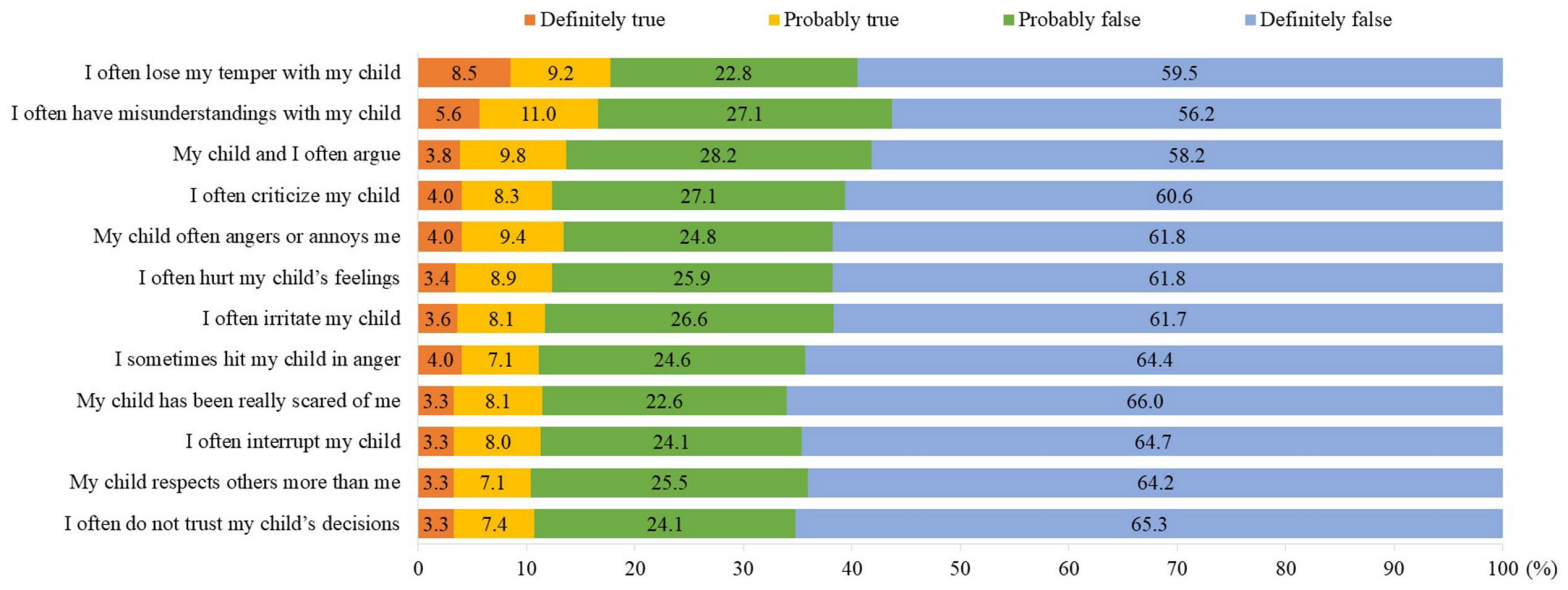
Responses for items in parental environment questionnaire (PEQ).

A total of 553 complete responses were received. The baseline characteristics of the study population are shown in the first and second columns of Table 1. There were almost equal responses from men (49.9%) and women (50.1%). The majority of parents (62.4%) were aged 50 to 59 years and of Malay ethnicity (64.0%). Over half reported an average household income of MYR2001–MYR3000. Regarding lifestyle factors, two-thirds (66.5%) reported sometimes/often engaging in physical exercise (37.6%) and 70.7% stated that they have practised healthy eating in the past 3 months. None reported consuming alcohol. Figure 1 shows the distribution of responses for the PEQ items. A total of 17.7% declared that it was *definitely true/probably true* that they often lose their temper with their children, followed by 16.6% who reported that it was *definitely true/probably true* that they often have misunderstandings with their children, and 13.6% said that it was *definitely true/probably true* that they often argue with their children.

**Table 1:** Factors associated with parent–child conflict (*N* = 553).

	Frequency (%)	Univariable analysis			Multivariable analysis
		Score 12–47 (*n* = 275)	Score 48 (*n* = 278)	*p*-value	Score 12–47 vs. score 48 OR (95% CI)
*Socio demographic characteristics*					
Age group (years)					
28–49	122 (22.1)	96 (78.7)	26 (21.3)	*p* < 0.001	1.84 (0.86–3.95)
50–59	345 (62.4)	115 (33.3)	230 (66.7)		0.34 (0.19–0.63)^**^
60–72	86 (15.6)	64 (74.4)	22 (25.6)		Reference
Gender					
Male	276 (49.9)	130 (47.1)	146 (52.9)	0.234	
Female	277 (50.1)	145 (52.3)	132 (47.7)		
Ethnicity					
Malay	354 (64.0)	220 (62.1)	134 (37.9)	*p* < 0.001	3.92 (2.14–7.19)^***^
Chinese	58 (10.5)	30 (51.7)	28 (48.3)		3.87 (1.74–8.64)^**^
Indian	140 (25.3)	25 (17.9)	115 (82.1)		Reference
Other	1 (0.2)	0 (0.0)	1 (100.0)		–
Marital status					
Married	507 (91.7)	266 (52.5)	241 (47.5)	*p* < 0.001	3.18 (1.30–7.75)^*^
Widowed/ Divorced/ Separated	46 (8.3)	9 (19.6)	37 (80.4)		Reference
Occupational type					
Professional and managerial	45 (8.1)	31 (68.9)	14 (31.1)	*p* < 0.001	0.61 (0.26–1.47)
Skilled worker	239 (43.2)	80 (33.5)	159 (66.5)		0.46 (0.27–0.77)^**^
Unskilled worker	117 (21.2)	75 (64.1)	42 (35.9)		1.45 (0.79–2.65)
Retired/ Unemployed/ Housewife	152 (27.5)	89 (58.6)	63 (41.4)		Reference
Average monthly household income (MYR)					
2000 and below	58 (10.5)	38 (65.5)	20 (34.5)	0.027	0.61 (0.26–1.47)
2001–3,000	321 (58.0)	149 (46.4)	172 (53.6)		0.82 (0.49–1.37)
3,001–5,000	174 (31.5)	88 (50.6)	86 (49.4)		Reference
Residence area					
Urban	535 (96.7)	260 (48.6)	275 (51.4)	0.004	Reference
Sub-urban	18 (3.3)	15 (83.3)	3 (16.7)		2.61 (0.62–11.02)
Lifestyle					
Doing physical exercises in the past 3 months					
Never/Seldom	185 (33.5)	141 (76.2)	44 (23.8)	*p* < 0.001	2.73 (1.49–5.01)^**^
Sometimes/	368 (66.5)	134 (36.4)	234 (63.6)		Reference
Often					
Practising healthy eating in the past 3 months					
Never/Seldom	162 (29.3)	123 (75.9)	39 (24.1)	*p* < 0.001	1.30 (0.68–2.48)
Sometimes/	391 (70.7)	152 (38.9)	239 (61.1)		Reference
Often					
Have enough sleep in a week in the past 3 months					
Never/Seldom	136 (24.6)	104 (76.5)	32 (23.5)	*p* < 0.001	1.85 (1.00–3.42)^*^
Sometimes/Often	417 (75.4)	171 (41.0)	246 (59.0)		Reference

^*^p < 0.05, ^**^p < 0.01, ^***^p < 0.001, Hosmer–Lemeshow test, chi-square: 37.603, *p* < 0.001, Nagelkerke *R*^2^: 0.452.

As shown in Table 1, the total PEQ score of the study participant range was 12 to 48, and the median PEQ score was 48.0 (interquartile range [IQR] 36 to 48). The PEQ score was categorised as 12–47 or 48, based on the median split; as such, a total of 275 (49.7, 95% CI 45.5–54.0) were categorised as having a score of 12–47 and 278 (50.3, 95% CI 46.0–54.5) were categorised as having a score of 48. Regarding demographics, for parents aged 60–72 years, the odds of having lower PEQ scores were significantly lower than those of parents aged 50–59 years (OR = 0.34, 95% CI 0.19–0.63). For ethnicity, the odds of having lower PEQ scores were higher amongst the Malay (OR = 3.92, 95% CI 2.14–7.19) and Chinese (OR = 3.87, 95% CI 1.74–8.64) respondents than amongst the Indian respondents. Parents who are skilled workers were found to have lower odds of having lower PED scores (OR = 0.46, 95% CI 0.27–0.77) than those not in employment (unemployed, housewife, or retired). Although there were no significant differences in the multivariate model, univariate analyses showed that households with a higher income reported a significantly lower level of parent–child conflict. The odds of having lower PEQ scores were significantly higher for married parents (OR = 3.18, 95% CI 1.30–7.75) than for widowed, divorced, or separated parents.

All three lifestyle factors were significantly associated with the level of PEQ scores in the univariate analyses. In the multivariate model, only physical exercise and adequate sleep remained significant. Parents who reported *never/seldom* engaging in physical exercise (OR = 2.73, 95% CI 1.49–5.01), practising healthy eating (OR = 1.30, 95% CI 0.68–2.48), and having enough sleep (OR = 1.85, 95% CI 1.00–3.42) were found to have higher odds of lower PEQ scores.

In total, only four parents were found to have symptoms of depression and stress, and six parents reported symptoms of anxiety. The proportions of parent–child conflict scores by symptoms of depression, anxiety, and stress are shown in Table 2. The proportions of depression, anxiety, and stress symptoms by demographic and lifestyle factors are shown in Appendix 2. The number of parents with symptoms of depression, anxiety, and stress was too small for important existing effects to be determined.

**Table 2:** Factors associated with depression, anxiety, and stress.

Psychological distress							
	Frequency (%)	Depression		Anxiety		Stress	
		Moderate/Severe/Extremely severe(*n* = 4)	*p*-value	Mild/Severe/ Extremely severe (*n* = 6)	*p*-value	Mild/Severe/ Extremely severe (*n* = 4)	*p*-value
*Parental environmental questionnaire*							
Total parent–child conflict score							
Low score (score 12–47)	275 (33.5)	3 (1.1)	0.308	5 (1.8)	0.106	3 (1.1)	0.308
High score (score 48)	278 (66.5)	4 (0.4)		1 (0.4)		4 (0.4)	

## 4. Discussion

The COVID-19 pandemic poses a threat to the well-being of children and families, particularly those in low-income communities. This study investigates the state of parent–child conflict and parents’ psychological health after the easing of social distancing. We hoped to gain insights into the potential long-term impact of the COVID-19 pandemic on family well-being. Understanding the impact of the pandemic on these outcomes is critical for developing resources and interventions for future pandemics as well as the provision of support for families in need.

The results revealed that only a minority of parents have been adversely affected by the pandemic. Despite a general low level of parent–child conflict, the minority of parents who exhibited a high level of parent–child conflict should not be overlooked as it is a key issue in family well-being and represents whole-family functioning. Without the pandemic, parent–child conflict is a normal part of family life and often escalates during the teenage years. The biological, psychological, and social changes occurring during adolescence may have a salient impact on parent–child relationships (28). Given that adolescents’ and parents’ reactions to pubertal development are common and may contribute to changes in family dynamics (29), interventions to promote healthy parent–child relationships should be a part of public health promotion, particularly at the height of the social restrictions imposed by a pandemic.

In this study, parent–child conflict appears to be more prevalent amongst parents of the oldest age range, 60–72 years, than amongst those aged 50–59 years. This implies that younger parents in this study are more likely to compromise in parent–child relationships. Intergeneration conflicts and tension between older parents and their children are not uncommon and have been reported in the past literature (30, 31). The results indicate a need for widespread family support and psycho-social interventions to reduce intergenerational conflicts, particularly during a pandemic lockdown. Our finding also suggests that older parents may benefit more from interventions.

An important insight of the study is that parents who were retired, unemployed, or housewives reported more parent–child conflict. Additionally, our study revealed a significant association between income and parent–child conflict in the univariate analysis. This could suggest the impact of economic pressure on family tension. Furthermore, an association between economic hardship and poor parent–adolescent relationships and intra-family conflict has been reported (32, 33). It has also been found that economic pressure is related to parental depressive symptoms, heightening couple conflict, which in turn results in harsh parenting and causes depressive symptoms in children (34). Our results indicate that hardcore poor households may need help to prevent or mitigate family-related conflicts.

Parent–child conflict and marital distress may coexist (35, 36). This perhaps explains the higher level of parent–child conflict amongst two-parent families in this study. There is mounting evidence showing that the COVID-19 pandemic and lockdown have led to greater conflicts and difficulties amongst couples (37–39). Notably, the COVID-19 quarantine has resulted in changes in marital life, exacerbated couple strains, and subsequently induced parent–child conflict. The findings suggest that psychological interventions geared towards promoting healthy couple relationships may hold promise for reducing mental strain in both parents and their children and ultimately enhance positive parent–child relationships.

Our findings indicate that practising healthy lifestyle behaviours has a positive effect on parent–child conflict reduction. Stress undermines health and well-being (40), and healthy lifestyle behaviours reduced people’s anxiety and sadness and improved their mental health during the COVID-19 pandemic (41). The study results may act as a basis for the promotion of family-centred intervention programs to encourage healthy behaviours in both parents and their children.

The current study found that there was generally a low prevalence of reported depression, anxiety, and stress symptoms amongst the sampled parents. The level of depression, anxiety, and stress symptoms found in this study were lower than comparable data from our previous study in Malaysia during the lockdown period (19). A more recent study found improvement in mental health amongst the public in Malaysia once the country was moving to ease the pandemic restrictions (42). Substantial relief from distress was also similarly found in Italy, and it was reported that end of strict lockdown, partial mitigation of preventive measures, relaunch of commercial, sport and school activities facilitate a psychological post-lockdown upswing in many people (43).

Despite a low level of psychological stress and parent–child conflict, the findings of this study imply that it is still crucial to maintain a healthy parent–child relationship after the pandemic. The Malaysian government has implemented various initiatives for low-income communities to alleviate the cost of living and burdens faced due to the COVID-19 pandemic. Amongst them were Bantuan Khas COVID-19 (BKC) cash aid assistance, income or employment loss assistance, and the implementation of financial relief in the form of loan moratorium or postponement of repayments for loans (44, 45). Low-income families are still continuously supported in recovering from the effects of COVID-19. This has perhaps brought positive benefits in relation to families’ financial burden and psychological well-being amongst the low-income groups. Despite a low level of depression, anxiety and stress, our findings provide key insights into the importance of continuous positive parenting and the need to reduce conflicts within the family and sustain healthy relationships even though normal economic activities and lifestyles have resumed.

There are some limitations to the current study that need to be considered when interpreting the results. Firstly, the cross-sectional design used could not infer a causal relationship. Secondly, the study sample represents a convenience sample of parents living in PPR houses. The key disadvantage of convenience sampling is that the sample lacks clear generalizability. Additionally, we only recruited parents from PPR houses in one state and federal territory in Malaysia; hence, the findings may not be generalised to the entire population of low-income housing residents in Malaysia. Future research should include a more representative sample. Additionally, the study relied on parents’ self-reports; therefore, socially desirable responses to sensitive questions in this study may be a source of bias leading to inaccurate self-reports and erroneous study conclusions. Finally, perhaps the most important limitation is the small sample in this study that reported symptoms of depression, anxiety, and stress; thus, the consequences of poor parent–child conflict for the mental well-being of parents were unable to be established. With the above-mentioned limitations, the findings of this study should be interpreted with caution. Despite the disadvantages, this study provides valuable data to fill the gap in the literature on the prevalence of parent–child conflict and its associated factors in low-income communities in Malaysia. This study also offers valuable information on the psychological effect of COVID-19 on the low-income population after the easing of the movement lockdown.

## 5. Conclusion

Based on data from our study sample, we found a low level of parent–child conflict and a small proportion of parents with severe to extremely severe anxiety, depression, and stress symptoms after the easing of the COVID-19 pandemic restrictions. Nonetheless, our study yielded several informative findings. In particular, it identified the socially vulnerable parent groups in low-income communities who should be the target for policies and services to overcome the aftermath of the COVID-19 pandemic and to equip them better in the event of future pandemics. Two-parent families, older parents, and parents who are economically disadvantaged are at higher risk of parent–child conflict. Establishing healthy lifestyle behaviours may be a key strategy in the prevention of parent–child conflict. In short, policies should take into consideration the implications of the lockdown for parents and their children. Provision of providing psycho-social intervention along with support to ease financial burden would be essential.

## Data availability statement

The raw data supporting the conclusions of this article will be made available by the authors, without undue reservation.

## Ethics statement

The studies involving human participants were reviewed and approved by this research was approved by the Universiti Malaya Research Ethics Committee (UM.TNC2/UMREC–1579). The patients/participants provided their online informed consent to participate in this study.

## Author contributions

LW, NF, SY, ZM, ZH, and YL contributed to concept and design and manuscript review. LW and HA contributed to literature search, data acquisition, and statistical analysis. The requirements for authorship as stated earlier in this document have been met and each author believes that the manuscript represents honest work. All authors contributed to the article and approved the submitted version.

## Funding

This study was supported by National Population and Family Development Board [Lembaga Penduduk dan Pembangunan Keluarga Negara (LPPKN)], The Ministry of Women, Family and Community Development Malaysia (GA031-2021)/(GPLPPKN0129) and Special Projects of the Central Government Guiding Local Science and Technology Development, China (No. 2021L3018).

## Conflict of interest

The authors declare that the research was conducted in the absence of any commercial or financial relationships that could be construed as a potential conflict of interest.

## Publisher’s note

All claims expressed in this article are solely those of the authors and do not necessarily represent those of their affiliated organizations, or those of the publisher, the editors and the reviewers. Any product that may be evaluated in this article, or claim that may be made by its manufacturer, is not guaranteed or endorsed by the publisher.
