# Supplementary material for: Parent–child relationships and psychological distress: survey of parents from low-income families after the COVID-19 pandemic
Source: Front Public Health. 2023 May 5;11:1158698. doi: 10.3389/fpubh.2023.1158698 (PMC10196452; doi:10.3389/fpubh.2023.1158698)
Supplement: Supplementary file 1 [file Table_1.DOCX]

Appendix 2 Factors associated with depression, anxiety and stress

|  | Frequency (%) | Depression | |  | Anxiety | |  | Stress | |
| --- | --- | --- | --- | --- | --- | --- | --- | --- | --- |
|  |  | Moderate/  Extremely severe  (n=4) | p-value |  | Mild/  Severe/ Extremely severe  (n=6) | p-value |  | Mild/  Severe/ Extremely severe  (n=4) | p-value |
| *Socio demographic characteristics* |  |  |  |  |  |  |  |  |  |
| Age group (years) |  |  |  |  |  |  |  |  |  |
| 28-49 | 122 (22.1) | 1 (0.8) | 0.095 |  | 2 (1.6) | 0.024 |  | 1 (0.8) | 0.095 |
| 50-59 | 345 (62.4) | 1 (0.3) |  |  | 1 (0.3) |  |  | 1 (0,3) |  |
| 60-72 | 86 (15.6) | 2 (2.3) |  |  | 3 (3.5) |  |  | 2 (2.3) |  |
| Gender |  |  |  |  |  |  |  |  |  |
| Male | 276 (49.9) | 1 (0.4) | 0.624 |  | 3 (1.1) | 1.000 |  | 1 (0.4) | 0.624 |
| Female | 277 (50.1) | 3 (1.1) |  |  | 3 (1.1) |  |  | 3 (1.1) |  |
| Ethnicity |  |  |  |  |  |  |  |  |  |
| Malay | 354 (64.0) | 4 (1.1) | 0.734 |  | 6 (1.7) | 0.348 |  | 4 (1.1) | 0.734 |
| Chinese | 58 (10.5) | 0 (0.0) |  |  | 0 (0.0) |  |  | 0 (0.0) |  |
| Indian | 140 (25.3) | 0 (0.0) |  |  | 0 (0.0) |  |  | 0 (0.0) |  |
| Other | 1 (0.2) | 0 (0.0) |  |  | 0 (0.0) |  |  | 0 (0.0) |  |
| Marital status |  |  |  |  |  |  |  |  |  |
| Married | 507 (91.7) | 4 (0.8) | 1.000 |  | 6 (1.2) | 1.000 |  | 4 (0.8) | 1.000 |
| Widowed/ Divorced/ Separated | 46 (8.3) | 0 (0.0) |  |  | 0 (0.0) |  |  | 0 (0.0) |  |
| Occupational type |  |  |  |  |  |  |  |  |  |
| Professional and managerial | 45 (8.1) | 0 (0.0) | 0.291 |  | 1 (2.2) | 0.283 |  | 0 (0.0) | 0.291 |
| Skilled worker | 239 (43.2) | 1 (0.4) |  |  | 1 (0.4) |  |  | 1 (0.4) |  |
| Unskilled worker | 117 (21.2) | 0 (0.0) |  |  | 1 (0.9) |  |  | 0 (0.0) |  |
| Retired/ Unemployed/ Housewife | 152 (27.5) | 3 (2.0) |  |  | 3 (2.0) |  |  | 3 (2.0) |  |
| Average monthly household income (MYR) |  |  |  |  |  |  |  |  |  |
| 2000 and below | 58 (10.5) | 1 (1.7) | 0.306 |  | 2 (3.4) | 0.192 |  | 1 (1.7) | 0.306 |
| 2001-3000 | 321 (58.0) | 3 (0.9) |  |  | 3 (0.9) |  |  | 3 (0.9) |  |
| 3001-5000 | 174 (31.5) | 0 (0.0) |  |  | 1 (0.6) |  |  | 0 (0.0) |  |
| Residence area |  |  |  |  |  |  |  |  |  |
| Urban | 535 (96.7) | 3 (0.6) | 0.124 |  | 4 (0.7) | 0.014 |  | 3 (0.6) | 0.124 |
| Sub-urban | 18 (3.3) | 1 (5.6) |  |  | 2 (11.1) |  |  | 1 (5.6) |  |
| *Lifestyle* |  |  |  |  |  |  |  |  |  |
| Doing physical exercises in the past 3 months |  |  |  |  |  |  |  |  |  |
| Never/Seldom | 185 (33.5) | 2 (1.1) | 0.605 |  | 4 (2.2) | 0.100 |  | 2 (1.1) | 0.605 |
| Sometimes | 368 (66.5) | 2 (0.5) |  |  | 2 (0.5) |  |  | 2 (0.5) |  |
| Often |  |  |  |  |  |  |  |  |  |
| Practicing healthy eating in the past 3 months |  |  |  |  |  |  |  |  |  |
| Never/Seldom | 162 (29.3) | 1 (0.6) | 1.000 |  | 3 (1.9) | 0.365 |  | 1 (0.6) | 1.000 |
| Sometimes | 391 (70.7) | 3 (0.8) |  |  | 3 (0.8) |  |  | 3 (0.8) |  |
| Often |  |  |  |  |  |  |  |  |  |
| Have enough sleep in a week in the past 3 months |  |  |  |  |  |  |  |  |  |
| Never/Seldom | 136 (24.6) | 1 (0.7) | 1.000 |  | 2 (1.5) | 0.639 |  | 1 (0.7) | 1.000 |
| Sometimes/ Often | 417 (75.4) | 3 (0.7) |  |  | 4 (1.0) |  |  | 3 (0.7) |  |
